# Epicardial Fat: Physiological, Pathological, and Therapeutic Implications

**DOI:** 10.1155/2016/1291537

**Published:** 2016-04-26

**Authors:** Juan Salazar, Eliana Luzardo, José Carlos Mejías, Joselyn Rojas, Antonio Ferreira, José Ramón Rivas-Ríos, Valmore Bermúdez

**Affiliations:** ^1^Endocrine and Metabolic Diseases Research Center, University of Zulia, Maracaibo 4004, Venezuela; ^2^Division of Pulmonary and Critical Care Medicine, Brigham and Women's Hospital and Harvard Medical School, Boston, MA 02115, USA; ^3^Internal Medicine Service, “Dr. Manuel Noriega Trigo” Hospital, San Francisco 4004, Venezuela

## Abstract

Epicardial fat is closely related to blood supply vessels, both anatomically and functionally, which is why any change in this adipose tissue's behavior is considered a potential risk factor for cardiovascular disease development. When proinflammatory adipokines are released from the epicardial fat, this can lead to a decrease in insulin sensitivity, low adiponectin production, and an increased proliferation of vascular smooth muscle cells. These adipokines move from one compartment to another by either transcellular passing or diffusion, thus having the ability to regulate cardiac muscle activity, a phenomenon called vasocrine regulation. The participation of these adipokines generates a state of persistent vasoconstriction, increased stiffness, and weakening of the coronary wall, consequently contributing to the formation of atherosclerotic plaques. Therefore, epicardial adipose tissue thickening should be considered a risk factor in the development of cardiovascular disease, a potential therapeutic target for cardiovascular pathology and a molecular point of contact for “endocrine-cardiology.”

## 1. Introduction

Once considered only as a mere storage compartment, the adipose tissue is now recognized for its extensive metabolic and endocrine functions [1, 2]. Adipose tissue is classified according to morphology, physiology, and embryological origin, and it is currently divided into two groups: white adipose tissue (WAT) and brown adipose tissue (BAT) [3]. The WAT derives from mesodermal stem cells [4] and is considered responsible for fat storage and energy reservoir. According to anatomy distribution, WAT is subcategorized as visceral and subcutaneous fat tissue [5]; the former is located within the muscle walls of the abdomen covering internal organs, whereas the latter is located under the skin, specifically in the hypodermis [6]. BAT originates from dermomyotome precursor cells [7], showing certain resemblance with skeletal muscle cell as they arise from similar pathways. This adipose tissue is located in small storage (contrary to WAT) and it has high vascularization and innervations, rendering a distinctive brown appearance. This tissue also metabolizes fat, produces heat, and contributes to the increase in overall metabolism [8–10].

Obesity is an inflammatory disease [11, 12] characterized by increase in the number and size of adipocytes, associated with progressive hypoxia, upregulation of proinflammatory cytokines, and chemotaxis of inflammatory cells. This phenomenon has been labeled as “adiposopathy” or sick adipose tissue [13]. According to various reports, a clear association between obesity and cardiovascular disease (CVD) has been observed [5, 14–16], relating to ectopic lipid storage, hyperglycemia, a procoagulant state, and an imbalance production of proinflammatory and anti-inflammatory adipokines, which mainly affect cardiovascular function [17]. In recent years, visceral adipose metabolism has proven to be important in the CVD development [18], indicating that each visceral body fat storage is anatomically and functionally different. Moreover, according to the closeness of the fat tissue to an organ, it exerts a specific local function for each one [19].

Epicardial fat is a WAT storage fat that covers 80% of the heart's surface, representing 20% of the organ's total weight [20]. Therefore, epicardial fat is considered to be a real VAT. This fat deposit is a major source of biomolecules and compartmentalized production of cytokines and hormones, acting as a localized gland [21]. Moreover, it regulates heart and blood vessel physiologically, via paracrine and vasocrine mechanisms. It has also been reported that epicardial adipose tissue (EAT) acts as an important energy reservoir for cardiomyocytes, which depend on fatty acid oxidation as energy source [14, 22]. Although EAT is needed for heart muscle function, in recent decades it has been published that increased thickness greatly enhances the risk of developing CVD and metabolic syndrome (MS) [23], becoming a new pharmacological target for primary and secondary prevention strategies.

## 2. Epicardial Fat: Morphology

EAT exhibits morphological similarities with the pericardial adipose tissue; however, it has a different embryological origin despite its anatomical proximity. Pericardial adipose tissue derives from the primitive thoracic mesenchyme, unlike EAT which originates from splanchnopleuric mesoderm [24]. Therefore, vascularization of both tissues is also different, where pericardiophrenic branches of the internal mammary artery supply blood for pericardial adipose tissue, while EAT is vascularized by coronary arteries [25]. EAT is mainly found in atrioventricular and interventricular grooves extending to the apex of the heart, specifically between the myocardium and visceral pericardium [26]. Adipose tissue storage in this anatomical area is divided into (a) pericoronary epicardial fat, which surrounds the adventitia of coronary arteries, myocardial, and (b) epicardial fat, located directly over the myocardium [27]. It is noteworthy to point out that these compartments are not separated by fascias or aponeurotic tissues, suggesting a close and strong interaction between the two structures, facilitating adipokines distribution into the heart muscle and arteries, and finally exerting a morphofunctional modulation in such organs [28].

## 3. Epicardial Fat: White, Brown, or “Beige” Adipose Tissue?

Embryologically, BAT is derived from myogenic progenitors expressing Myf5 (encoding myogenic factor 5) and Pax7 [29], while “beige” adipose tissue has been arising from the transdifferentiation of mature cells, as well as Myf5^−^ precursors and recently MYH11^+^. This diversity of origins is a reason for research in animal models [30]. It is well known that BAT generates heat in response to cold temperatures and autonomic nervous system activation, related to a high number of mitochondria and uncoupling proteins production [31]. EAT, despite being phenotypically similar to WAT, highly expresses uncoupling protein-1 (UCP-1, OMIM 113730) in their membranes [32, 33] suggesting that it could function similarly to the BAT (i.e., heat production). These biological and functional changes have warranted the application of a new name for these* transdifferentiated* adipose cells, the “beige” adipocyte [34], which are related to prolonged exposure to cold weather and *β*-adrenergic agonist and whose characterization has been done in the last decade [35].

Continuing with this line of thought, UCP-1 works as a proton translocator in the inner mitochondrial membrane, producing proton flow into the mitochondrial matrix, decreased production of ATP, and energy dissipation in the form of heat [36]. Furthermore, EAT also expresses activating receptors of peroxisome proliferator *γ* coactivator 1*α* (PPAR*γ*C1*α*, OMIM 604517), which is one of the most important proteins in the differentiation of adipocytes [7]. In cases of hypothermia, chronic exposure to cold promotes PPAR*γ*C1*α* activation, meaning that epicardial fat may protect the myocardium by stimulation of the white-to-beige adipocyte transformation [4, 34, 37], avoiding the development of ventricular arrhythmias and thus exerting a cardioprotective role [32]. Taking this information into account, EAT then acts as a local energy source in cases of high cardiovascular demand as in ischemic conditions [38].

## 4. Epicardial Fat: An Endocrine Organ

As previously mentioned, adipose tissue is not only a simple lipid storage unit; it also serves as an endocrine and paracrine organ, having a key role in maintaining the homeostasis of body energy, lipids, and carbohydrates metabolism [39, 40]. EAT is a metabolically active organ and a major source of anti-inflammatory and proinflammatory adipokines [41–43] (see Table 1), which have significant impact on cardiac function and morphology [27, 44]. Likewise, numerous cell groups including mainly inflammatory cells such as lymphocytes (CD3^−^), macrophages (CD68^−^), and mast cells [45] have been identified as resident cells in EAT. Hirata et al. [46] have shown that the ratio of M1/M2 macrophages in EAT correlates with the severity of coronary artery disease. Therefore the polarization of the macrophage can be of significant importance in the epicardial fat inflammatory phenomenon.

### 4.1. Anti-Inflammatory Adipokines

Anti-inflammatory cytokines contribute to the regulation of vascular tone and maintain blood pressure and proper functionality of cardiac contractility [47, 48]. The most important adipokines expressed in epicardial tissue are adiponectin, adrenomedullin, and omentin [49].

Adiponectin (OMIM 605 441) is a peptide hormone with 247 amino acids, which is produced only by adipose cells, described to have antidiabetic, antiatherogenic, antioxidative, and anti-inflammatory properties [65, 66]. There are two types of receptors for this hormone, Adipo-R1 and Adipo-R2, which are expressed only in insulin sensitive tissues [67]. These are responsible for increasing the expression of molecules involved in the fatty acid oxidation and lipid mobilization, leading to increased TAG turnover, lowering lipid deposit in tissues. Intracellular lipid droplets have been known to interfere with the activation of phosphatidylinositol 3-kinase (PI3K) by insulin, and, therefore, with the translocation of the glucose transporter 4 (GLUT-4) to the plasma, thus preventing subsequently the entry of glucose into the cardiomyocytes membrane [50]. In addition, adiponectin also has an insulin sensitizing effect via increased fatty acid oxidation mediated by AMP-activated protein kinase (AMPK) and inhibiting acetyl-CoA carboxylase in cardiac muscle [66, 68, 69]. Remarkably, this adipokine also inhibits the production of tumor necrosis factor alpha (TNF-*α*) and other inflammatory pathways in adipocytes and macrophages, producing an anti-inflammatory effect [70]. The interaction of adiponectin with its receptor Adipo-R2 on the endothelial surface induces nitric oxide (NO) production and blunts platelet aggregation, leukocyte adhesion to endothelial cells, and vascular smooth muscle proliferation [71], the very defenses against endothelial dysfunction development [72].

Furthermore, EAT is also responsible for high quantity release of adrenomedullin (OMIM 103275), a potent vasodilator peptide with 52 amino acid residues which is produced in a variety of peripheral organs, mainly in kidneys, lungs, adrenal glands, adipocytes, and cardiovascular system [51]. This peptide exerts its action by interacting with the calcitonin receptor-like receptor (CRLR), belonging to the family of G-coupled protein receptors [73]. In cardiomyocytes, adrenomedullin is responsible for activating adenylate cyclase, increasing cytosolic cAMP and activation of protein kinase A (PKA) [52], inducing various downstream effects including increase in intracellular calcium [53–55]. PKA stimulates calcium voltage-gated channels located in the sarcoplasmic reticulum of cardiomyocytes, causing a conformational change in its structure and release of calcium into the cytosol, which ultimately contributes to an increase in cardiac output [55, 56]. In the bloodstream, adrenomedullin is able to inhibit the migration and proliferation of vascular smooth muscle cells and also inhibits apoptosis of endothelial cells and the production of endothelin-1 (ET-1, OMIM 131240) [57]. It has been proposed that adrenomedullin has antioxidant properties, capable of antagonizing oxidative stress and reactive oxygen species (ROS) induced by angiotensin II [58].

### 4.2. Proinflammatory Adipokines

The release of proinflammatory cytokines by the EAT relates to innate inflammatory response, which can be activated through Toll-like receptors (TLRs) located in the cell membrane of macrophages, B cells, dendritic cells, and, until recently, adipocyte membranes [59, 74]. TLRs recognize antigens such as lipopolysaccharide (LPS) and saturated fatty acids, which act as endogenous ligands in adipose tissue [74], enhancing nuclear factor kappa beta (NF-*κβ*) translocation into the nucleus of epicardial adipocytes with subsequent transcription of inflammatory mediators such as interleukin 1 (IL-1), interleukin 8 (IL-8), interleukin 6 (IL-6), and TNF-*α*, thereby interconnecting innate immunity and chronic inflammation in obese individuals [60–62].

In relation to IL-6 (OMIM 147620), this is a glycosylated protein that is mainly secreted by VAT [75]. It can act via paracrine, autocrine, and endocrine mechanisms, participating in body weight control and energy homeostasis [76]. This cytokine induces vascular smooth muscle proliferation, one of the key features in atherosclerotic plaques [63], functioning as an important component in endothelial dysfunction. In the same manner, it produces the inhibition of gene expression of adiponectin, which contributes to the exacerbation of hypertension associated with obesity. Similarly, it has been published that IL-6 is involved in insulin resistance, altering signaling in hepatocytes by induction of SOCS-3 protein, thereby inhibiting the autophosphorylation of the insulin receptor, stimulating gluconeogenesis and hepatic secretion of TAG [49, 64]. Ridker et al. [77] conducted a study with 14,916 apparently healthy men, measuring fasting IL-6 plasma levels during a 6-year period. They reported that as blood pressure increased, IL-6 levels and associated cardiovascular mortality increased, concluding that IL-6 plasma levels could be used as predictors of future myocardial infarction [77].

Another proinflammatory cytokine secreted by epicardial fat, which is also elevated in obesity and actually worsens insulin resistance, is TNF-*α* (OMIM 191160). This cytokine is a potent vasoconstrictor, which oddly can also exert vasodilation in a dose-dependent manner via NO and prostaglandin production, confirming that the mechanisms of vascular tone control by this cytokine are still not completely understood [78]. Vasoconstriction induced by TNF-*α* is associated with increased production of angiotensin II and ET-1 [79]. In a metabolic level, TNF-*α* also induces lipolysis and thereby activates mitogen activated protein kinase (MAPK), decreasing the activity of the insulin receptor substrate 1 (IRS-1) by phosphorylation of serine residues, finally inhibiting GLUT-4 expression [80]. In addition, TNF-*α* decreases adiponectin secretion and stimulates production of other proinflammatory substances such as IL-6, thus contributing to the maintenance of chronic inflammatory of adipose tissue observed in obesity [81].

## 5. Epicardial Fat and Vasocrine Regulation

**Figure 1:**
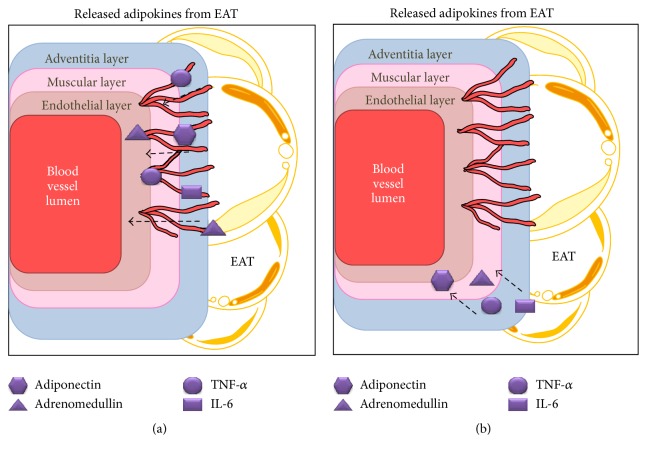
EAT secretion mechanism. Adipokines entering the wall by a “vasocrine” mechanism (a) using as transportation vasa vasorum or using a paracrine mechanism (b), which through dissemination adipokines pass down the gradient of concentration. The molecules continue to step up to the deeper layers of blood vessels (endothelial and muscular) which trigger several effects. EAT: epicardial adipose tissue, TNF-*α*: tumoral necrosis factor alpha, and IL-6: interleukin 6.

By releasing various molecules, epicardial fat tissue is capable of regulating vascular tone, by variation in the size of their diameters [82]. The entry of molecules into the vessel wall will depend on its thickness, so molecules from adipose tissue diffuse into the wall of the medium and larger caliber arteries through a mechanism known as “*vasocrine*” [83, 84], using* the vasa vasorum* in the adventitia layer as an entry point (Figure 1). Moreover, this vasocrine mechanism does not apply to smaller arteries because their walls are thinner. When molecules from adipocytes enter into the vessel walls through diffusion, it is known as a paracrine mechanism [85]. The perivascular adipose tissue that conforms to EAT releases adipokines and hormones that affect coronary blood vessels, through the mechanisms mentioned above, causing a “vasocrine regulation” (VR) in the coronary arteries [83, 86]. This regulation is about the change in diameter of the vessels from the molecules secreted by the EAT. Communication between the molecules secreted by adipose tissue and blood vessels is fundamental for the proper vascular functioning and they can vary according to the scenario, contributing to vascular homeostasis or in pathological cases promoting vascular disease [87].

### 5.1. Vasocrine Regulation under Physiological Conditions

**Figure 2:**
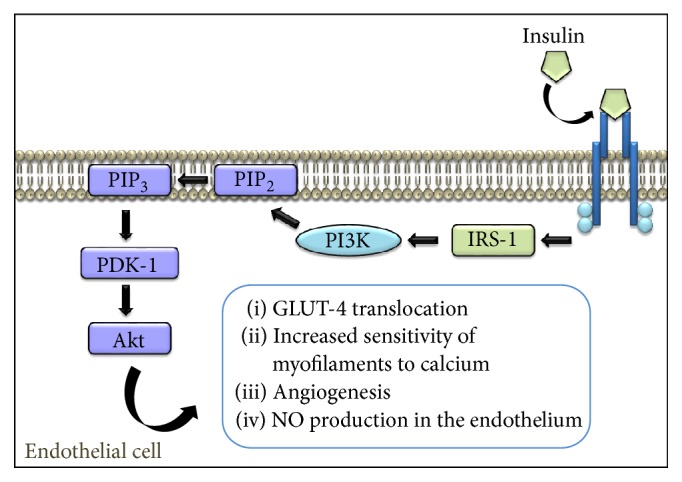
Mechanism of action of insulin in the endothelial cell. The interaction of insulin with its receptor results in autophosphorylation of tyrosine residues and heterophosphorylation of second messengers such as IRS-1, which phosphorylates PI3K, leading to the activation of a cascade of phosphorylation ending with expression of various physiological effects, like the production of endothelial NO. IRS-1: insulin receptor substrate 1, PI3K: phosphatidylinositol 3-kinase, PIP_2_: phosphatidylinositol diphosphate, PIP_3_: phosphatidylinositol triphosphate, PDK-1: dependent kinase PI3K, Akt: protein kinase B, GLUT-4: glucose transporter 4, and NO: nitric oxide.

Hyperglycemia is a stimulus for the synthesis of insulin by the beta cells of the pancreas, located in the Langerhans islets [88]. After insulin is secreted through the portal venous system, 50% is degraded after liver passage [89]; the other 50% reaches the systemic circulation, where it binds to its receptors on the cell membrane of the target organs, triggering a cascade of intracellular second messengers [90]. Inside the endothelial cell, the first molecule to interact with the insulin receptor is IRS-1 (insulin receptor substrate 1), which has multiple tyrosine residues [91, 92]. The pathway related to VR is the PI3K pathway, very important during cell growth and survival and especially in modulation of vessel diameter by NO production. The signaling cascade starts with the binding of the enzyme with IRS-1, resulting in PI3K activation and subsequent production of phosphatidylinositol triphosphate (PIP_3_) from phosphatidylinositol diphosphate (PIP_2_). PIP_3_ activates PI3K-dependent kinase (PDK-1) which in turn phosphorylates protein kinase B (Akt) [93, 94]. Then, Akt promotes GLUT-4 translocation, allowing the passage of glucose into muscle and adipose tissue, producing an increased calcium sensitivity in myofilaments, improving muscle cell contraction, and stimulating angiogenesis and the production of NO in the endothelium (Figure 2) [95].

Although the physiological role of EAT on vessels is not entirely clear, it has been shown that epicardial adipocyte through the secretion of adiponectin and adrenomedullin exerts anti-inflammatory, antiatherogenic, and even antidiabetic effects in coronary arteries [96, 97], participating in the regulation of the diameter on the vessel wall. The binding of adiponectin to its receptors, AdipoR1 and AdipoR2, causes an interaction with the APPL1 protein, which, according to a study by Cheng et al. [98], could function as a signaling cascade of events that promote the production of NO via eNOS, working as an intermediary between adiponectin receptors and AMPK, attenuating the vascular muscle cells migration and subsequent weakening of the vessel wall [99]. Moreover, adiponectin stimulates cAMP and PKA pathways, which suppress NF-*κβ*, blunting proinflammatory signaling mediated by TNF-*α* and inhibiting cell adhesion molecules expression and mobilization towards the cell membrane [100].

### 5.2. Vasocrine Regulation in Pathological Conditions

Due to the known plasticity of adipocytes observed in their preadipocyte differentiation into macrophages and hypertrophy/hyperplasia of differentiated adipocytes, epicardial adipocyte undergoes various changes as well [70, 101]. These morphofunctional changes generate hypersecretion of proinflammatory and proatherogenic adipocytokines, associated with decreased production of adiponectin and adrenomedullin [101, 102]. One of the changes observed in enlarged adipocytes is an increased production of saturated free fatty acids (FFA), which bind to TLR-4 in macrophages resulting in the activation of NF-*κβ* and enhanced synthesis of TNF-*α* [103, 104]. TNF-*α* activates macrophages derived from* transdifferentiated* adipocytes, inducing lipolysis and increasing the expression of several genes, such as intracellular adhesion molecule-1 (ICAM-1), IL-6, and monocyte chemoattractant protein-1 (MCP-1) [105, 106]. However, macrophages are not only derived from the differentiation of preadipocytes, but they can also arise from monocytes that diffuse through the subendothelial space via ICAM-1 and MCP-1 [107]. This local paracrine system that involves FFA adipocyte release and TNF-*α*-induced macrophages establishes a cycle that leads to a constant proinflammatory state or adiposopathy [82, 86, 108].

Blood vessels express receptors for the majority of adipokines derived from adipose tissue, playing an important role in cardiovascular pathophysiology [108]. The proximity between the EAT and coronary blood vessels makes proinflammatory adipokines from epicardial fat to the vascular wall much easier and efficient [109]. An increased diffusion of IL-6 and TNF-*α*, mainly in the vascular wall, decreases tissue sensitivity to insulin by inhibiting one of its metabolic pathways [70]. During VR, TNF-*α* from EAT easily diffuses to blood vessels inhibiting the PI3K pathway in endothelial cells via the TNF-*α* receptor 1 (TNF-R1), which has the serine-threonine kinase activity that is responsible for phosphorylating serine and threonine residues of IRS-1, thereby preventing phosphorylation cascade [110, 111]. At the same time, TNF-R1 starts its own signaling associated with the RIP1-TRADD-TRAF2 complex, formed by the associated death domain receptor of TNF-R1 (TRADD), protein-1 that interacts with the receptor (RIP1), and factor 2 associated with TNF-R1 (TRAF2). Immediately after the assembly of the complex, NF-*κβ* inhibitors are phosphorylated and inhibited, permitting the translocation of this transcription factor to the nucleus, where it finally binds to specific sequences of DNA and induces the expression of genes involved in the synthesis of proteins related to inflammation, autoimmunity, immune maturation, and adaptive response [111–113].

Besides contributing to the maintenance of chronic inflammatory of EAT and insulin resistance in the endothelial cells, it has been shown that an increase in TNF-*α* locally promotes vasoconstriction associated with the production of ET-1 in the endothelium of the coronary arteries, which may be enhanced in cases of insulin resistance [114]. The MAPK pathway plays an important role in modulating gene transcription, being responsible through its signaling cascade for the activation of the gene encoding for ET-1 [115]. This pathway activated by TNF-*α* promotes the phosphorylation of extracellular signal-regulated kinase (ERK-1) which goes from the cytosol to the nucleus and induces the expression of ET-1 [116, 117]. Overall, 75% of the resulting ET-1 is mobilized into the vascular smooth muscle cells close to the endothelium via diffusion, joining the EAT receptor responsible for mediating vasoconstriction [118]. Stimulation of these receptors, G-coupled protein receptors, by the ET-1 activates phospholipase C, inducing its mobilization to the cell membrane to hydrolyze PIP_2_ and generate diacylglycerol and inositol triphosphate [119, 120]. Diacylglycerol works as a mediator in the intracellular communication system and increases the activity of protein kinase C (PKC). It also phosphorylates various proteins that control cell proliferation. Simultaneously, inositol triphosphate mobilizes Ca^2+^ stored in the sarcoplasmic reticulum into the sarcoplasm causing an increase in cytosolic levels inducing vasoconstriction of coronary arteries [121].

Taking all these mechanisms into consideration, the potential role of endocrine regulation is evident in vascular disease mediated by proinflammatory factors secreted in the underlying adipose tissue, at least in the molecular level [83]. Nowadays, there are only reports showing a relationship between synthesized mediators, the control of vascular tone, and insulin sensitivity in vitro models [84, 122]. New studies are needed that quantify the probability of risk in the occurrence of cardiovascular diseases and type 2 diabetes mellitus and for each biomolecule synthetized in the perivascular adipose tissue, as well as comparing the effect when these biomolecules mediated an endocrine, paracrine, or vasocrine effect.

## 6. Epicardial Fat: How to Quantify It?

Increasing VAT is associated with increased CVD and metabolic syndrome risk, so an epicardial adiposity thickening can be considered as a marker for heart disease. In this line of thought, EAT quantification would allow its use as a potential therapeutic target [109, 123, 124]. Previous studies have shown interest in the association between EAT and the increase in ventricular mass, noticing compensatory hypertrophy of the left ventricle (LV) in response to pressure or volume overload combined with hormonal effects. LV hypertrophy is proportional to the increase of epicardial fat, which has been established by measuring them by transthoracic echocardiography [26, 124, 125]. A clinical study from Kim et al. [126] using this technique evaluated 27 individuals at baseline and after they were subjected to a hypocaloric diet (with a reduction of 26.8% of the daily caloric intake) and an aerobic exercise program for a period of 12 weeks, publishing a final reduction of 17.2% EAT volume at the end of the study. Meanwhile, Salami et al. [127] investigated whether there was a difference in EAT thickness among white and black men who were admitted for chest pain symptoms. The team performed transthoracic echocardiography in 150 patients, reporting that the EAT in the right ventricle was significantly higher in white than in black men, concluding that it can be considered an important variable when analyzing the relationship between fatty storage and cardiovascular risk factors.

According to the studies mentioned above, the primary method for epicardial fat measurement is the standard dimensional transthoracic echocardiography (2D) which is a safe, easily reproducible, and noninvasive method that can be routinely done in patients with suspected cardiovascular or metabolic syndrome risk [21, 109]. For this procedure, the parasternal long and short axis views in 2D are used to achieve a more accurate measurement of fat thickness in the right ventricle (RV). The thickness is measured perpendicularly to the RV free wall at the end of systole in 3 cardiac cycles, and this is because during diastole epicardial fat is compressed, giving little accurate measurements [128].

Despite the advantages, echocardiography is not an optimal technique for the quantification of EAT since it does not reflect its total volume [28, 129]. Other imaging methods used for epicardial fat quantification are multislice computed tomography (MCT) and magnetic resonance (MR), considered as the ultimate tests to evaluate EAT between the myocardium and visceral pericardium [28]. With MCT volume measurement (3D) is possible, as well as obtaining information regarding coronary artery calcification and evaluation of stenosis. This technique has major disadvantages including exposure to ionizing radiation and especially its high cost [130–132]. MRI has better spatial resolution and volumetric assessment of adipose tissue but differs from MCT due to lack of radiation exposure. However, it is also expensive and has higher time consumption during the procedure, giving it a significant disadvantage [132, 133]. In this sense, Tachibana et al. [134] have evaluated the predictive ability of transthoracic echocardiography to predict high-risk plaques, confirmed by coronary computed tomography angiography from epicardial fat thickness, with this being considered a good noninvasive predictor. Therefore, the use of echocardiography is the most cost-effective method today especially in regions where the availability of high cost imaging studies is low.

## 7. Epicardial Fat in Clinical Practice

The relationship between the increase of EAT and CVD is now considered a common discussion subject, being especially associated with coronary syndromes and weakening of atheromatous plaques [135, 136]. Ito et al. [135] conducted a study in 117 patients which were evaluated for epicardial fat volume through MCT and the inside of the arteries was assessed by optical coherence tomography to detect thin fibroatheromatous layers, noting that, with increasing volume in EAT, plates were becoming thinner and susceptible to their break. In another study conducted by Alexopoulos et al. [137], 214 patients between 40 and 68 years were evaluated, observing that as the severity of the stenosis of the vessel progressed EAT volume progressed and that it was significantly greater in patients who had mixed and noncalcified plaques than those who had calcified or atheromatous plaques. Meanwhile, Mahabadi et al. [138] determined the predictive ability of EAT for coronary events under the Heinz Nixdorf Recall study, a population-based study in more than 4000 German individuals, in which it was observed that the volume of EAT had a significant association with the occurrence of coronary events, independently of traditional cardiovascular risk factors. This was an independent association in spite of the presence of calcified coronary arteries, which suggest that fat deposits may be related to future cardiovascular events through a different pathway.

Also, Okada et al. [139] analyzed the relationship between the volume of EAT and the severity of coronary artery disease in nonobese patients as well as the potential effect that the epicardial fat volume has on coronary plaque morphology or the extension thereof. Similarly to the previous study, individuals with increased volume of EAT had a higher severity in coronary level plaques, which were not necessarily calcified, indicating that the thickness storage of epicardial fat has a key role in the progression of coronary atherosclerotic disease, even in those individuals with no accumulation of visceral fat. In addition, correlations between increased EAT and reduced HDL-C and increased IL-6 and high sensitivity C-reactive protein (hs-CRP) were observed.

From a clinical standpoint, EAT thickening can be considered a potential risk factor that would appear in the early stages of coronary plaques formation and their vulnerability phases [140–142]. The plaque rupture arises in the fibrous layer, which is usually thin and heavily infiltrated by macrophages, which are responsible for maintaining a proinflammatory state of the underlying tissue. Furthermore, EAT secretion of TNF-*α*, IL-1, and MCP-1 stimulates macrophages and induces apoptosis of vascular smooth muscle cells [143, 144]; both events might contribute to weakening of the fibrous layer and subsequent cleavage of the plate. However, the precise mechanisms by which the breaking of the plaque occurs have not yet been fully understood [144].

As for MS, the diversity of risk factors has been linked to the storage of visceral dysfunctional adipose tissue [150]. In this regard, Iacobellis et al. [145] were the first to link the increase of EAT and the presence of the various components of MS, especially abdominal obesity in 72 individuals undergoing echocardiography. Also Yorgun et al. [146] have shown that the EAT is related to age, BMI, and the various components of MS in 83 patients who underwent multidetector computed tomography. Meanwhile, Okyay et al. [147] suggest that the relationship is with the thickness of subepicardial adipose tissue in patients with a diagnosis of MS. However, these findings are not limited only to the adult population; in a study of individuals over 65 years, Kaya et al. [148] considered EAT thickening as a diagnostic criterion being observed in the geriatric population, with a predictive power of 90%. In accordance with the age group, Fernández Muñoz et al. [149] showed similar findings in Mexican postmenopausal patients, but the sample sizes of these studies preclude the generalization of results. Also Akyol et al. [151] have observed in teenagers with obesity and MS a close relationship between EAT, the thickness of the carotid intima, and early cardiac dysfunction parameters showing the predictive role of lipid accumulation for the adult and elderly population (Table 2).

Despite multiple reports that posed a close relationship between EAT thickness and presence of coronary disease, there are not current specific recommendations for its use in a particular group of patients due to the heterogeneity in the early studies results which agree with the echocardiography effectiveness in predicting coronary artery disease [152–154]. However, Picard et al. [155] have found a higher correlation with the severity of coronary disease by angiography quantified in patients with chest pain. That is why we consider important focus of future research to determine which group of patients would benefit from routine analysis of epicardial fat, especially those classified in intermediate stages of cardiovascular risk based on ethnic differences such as the fact that it has been raised in connection with MS [156].

## 8. Sick Epicardial Adipose Tissue: Potential Pharmacological Interventions

Once the detailed mechanisms associated with EAT were described in scientific literature, the pharmacological potential of this tissue was established. Several strategies of treatment have been implemented, either associated with lipid metabolism or mainly related to glucose homeostasis.

Hydroxymethyl glutaryl-CoA reductase inhibitors, better known as statins, are responsible for limiting cholesterol biosynthesis due to blockage of the HMG-CoA to mevalonic acid step, and they increase the expression and activity of LDL receptors [162]. Furthermore, statins have other pleiotropic effects as inhibiting the growth of macrophages, secretion of metalloproteinases, and inhibition of cell adhesion in atherosclerosis, being categorized as plaque stabilizers [162–164]. Park et al. [157] conducted a study with 145 patients for a period of two years, where 82 individuals took 20 mg of atorvastatin while the other individuals took 10 mg simvastatin combined with 10 mg of ezetimibe; these individuals were constantly evaluated with 2D transthoracic echocardiography for EAT assessment. The study showed a statistically significant decrease in the concentration of total cholesterol, TAG, LDL, and epicardial fat storage with the continued use of these drugs. Atorvastatin and simvastatin/ezetimibe showed similar effects on lipids, but it was the atorvastatin that produced a significantly greater decrease in the thickness of EAT, although it is unknown what caused this difference between these 2 drugs. Likewise, treatment with statins as well as thickness of EAT has been linked to maintenance of sinus rhythm in patients with atrial fibrillation after electrical cardioversion [165]. Statin therapy and thinner EAT are associated with a better heart rate response, an effect that may be related to the inflammatory role of this tissue [166]. Similarly, treatment with pioglitazone has been associated with genetic modulation in the expression of proinflammatory cytokines in patients with coronary artery disease and type 2 diabetes mellitus compared to healthy individuals [158].

Moreover, Lima-Martínez et al. [159] recently published their work concerning the effect of sitagliptin over EAT in type 2 diabetes patients. This pilot study was realized as a 24-week interventional study with 26 patients with average HbA1c ≥ 7% with ongoing metformin monotherapy. Sitagliptin was added using a fixed combination of 50/1000 mg tablet given twice a day. EAT was measured via ultrasound evaluation at baseline and after 24 weeks, reporting significant reduction in EAT volume. It has also recently proposed a similar effect of Liraglutide in reducing fat at the cardiac region independently of the body weight loss [167].

Additionally, Elisha et al. [160] reported that insulin replacement therapy could be associated with increased EAT mass, as shown by their open label interventional study comparing insulin detemir with insulin glargine. This team published that insulin detemir resulted in less fat thickening at 24 weeks, associated with less weight gain and less truncal lean mass loss. This suggests that EAT could be used as an indirect marker for visceral adipose tissue and as a valid target for therapy; hence pharmacological therapy and prognosis should take into account status of EAT, especially in those with higher coronary risk. It is also important to mention the fact that not only have pharmacological measures been associated with a decrease in the volume of EAT, but also both physical activity and nutritional changes have shown a considerable effect on this tissue [161, 168] (Table 3).

## 9. Conclusions

The EAT is a primary source of biomolecules that by diffusion or vasocrine mechanisms enter into the blood vessel wall, considered a potential cardiovascular risk factor especially in obesity. The proximity of epicardial fat deposit with the myocardium and its irrigation coming from the coronary arteries indicates that although EAT is not as abundant in quantity as other body fat deposits, it drastically influences the constitution of the muscular wall of the heart and its vessels. Therefore, the measurement of epicardial fat deposition through 2D transthoracic echocardiography is important since it is the most accessible and accurate method. In terms of modulation, the use of statins could function as a possible treatment to help decrease the volume of EAT and contribute to the stabilization of atherosclerotic plaques. It is essential to continue with the investigation of the associated adipokines and mechanisms involved in this pathophysiology, including the proteomic and transcriptome analysis of this tissue [168], due to the high prevalence of cardiovascular and metabolic disorders.

## Figures and Tables

**Table 1:** Main adipokines release by EAT and other tissues.

Adipokines	Production tissue	Mainly associated function	References
Anti-inflammatory			
Adiponectin	Adipose tissue	Oxidation and transport of fatty acid	[49, 50]
Adrenomedullin	Adipose tissue Adrenal medulla Heart Lung Kidney	Increase in cytosolic Ca^2+^ ET-1 inhibition NF-*κβ* inhibition	[51–58]
Omentin	Adipose tissue (*specially in epicardial fat*)	Akt-phosphorylation in isolated blood vessels, vascular smooth muscle cells, and microvascular endothelial cells Enhancing insulin-mediated Akt-phosphorylation and glucose uptake in adipocytes	[41]

Proinflammatory			
TNF-*α*	Adipose tissue Immune system cell	Cellular proliferation NF-*κβ* translocation Inhibition of adiponectin secretion Lipolysis induction	[59–64]
IL-6			
IL-1			
IL-8			
Resistin	Adipose tissue Blood mononuclear cells Macrophages	Insulin resistance Angiogenesis Thrombosis Vascular smooth muscle cell migration and proliferation	[42]
Visfatin	Adipose tissue	Cell proliferation Monocyte/macrophage activation and recruitment Vascular inflammation and remodeling Insulin-mimetic Fat-secreted factor?	[43]

**Table 2:** Studies of epicardial fat as a factor associated with coronary events and metabolic syndrome.

	Author (reference)	Methodology	Conclusions
Coronary events	Ito et al. [135]	Study in 117 patients with simple coronary lesions underwent MCT	EFV was associated with plaque vulnerability, being an independent predictor of ACS (OR: 2.89; 95% CI: 1.14–7.29); *p* = 0.025
	Alexopoulos et al. [137]	Study in 214 patients that underwent contrast-enhanced CT angiography images without a history of PCI, coronary artery by-pass surgery, or cardiomyopathy	There is an increase in EAT volume in patients with CAD, being an independent predictor of noncalcified plaques (OR: 3.85; 95% CI: 1.42–10.45); *p* < 0.01
	Mahabadi et al. [138]	4093 randomly selected participants under the *Heinz Nixdorf Recall Study *were prospectively evaluated; epicardial fat was assessed by cardiac CT	Epicardial fat is associated with the occurrence of fatal and nonfatal coronary events, regardless of the presence of other risk factors and coronary artery calcification score (HR: 1.50; 95% CI: 1.07–2.11); *p* = 0.02
	Okada et al. [139]	Study in 140 patients with chest pain with known or suspected arterial disease who were not obese and underwent sixty-four MCT	EFV is associated with severity of CAD and with the presence of noncalcified or mixed plaques (without plaques: 85.0 ± 4.2 mL; with nonobstructive plaque: 91.0 ± 8.8 mL; with obstructive plaque in a single vessel: 94.8 ± 6.8 mL; with obstructive plaque in left main or multiple vessels: 105.7 ± 7.3 mL; *p* < 0.05)

Metabolic syndrome	Iacobellis et al. [145]	Study in 72 consecutive patients with BMI between 22 and 47 kg/m^2^; each patient underwent two-dimensional (2D) guided M-mode TTE	MRI VAT is best related to EAT compared to abdominal circumference and thus should be considered an indicator of VAT and cardiovascular risk. Also abdominal circumference was the component more related to MS (*r* ^2^ = 0.428; *p* = 0.01)
	Yorgun et al. [146]	Study in 83 patients with suspected CAD who underwent dual source MDTM	Both EAT and the thickness of the pericoronary fat were associated with the presence of MS; they could be considered useful indicators for this disorder (*β* = 7.80; *t* = 2.42; *p* = 0.018)
	Okyay et al. [147]	Case-control study in 246 patients (123 with MS and 123 without MS) who underwent M-mode TTE.	There is a close association between subepicardial adipose tissue and the presence of MS, its measurement being a feasible method for evaluating the MS and cardiovascular risk (*β* = 0.158; *t* = 3.019; *p* = 0.003)
	Kaya et al. [148]	Case-control study in 60 patients (30 with MS and 30 without MS) over 65 years old who were subjected to two-dimensional echocardiographic method by TTE	EAT was higher in geriatric patients with MS; a value of 7.3 mm or more showed high sensitivity and specificity in predicting MS and it could be considered a diagnostic criterion (*β* = 17.35; Wald = 12.36; *p* < 0.001)
	Fernández Muñoz et al. [149]	Cross-sectional study in 34 postmenopausal women with and without MS who underwent TTE	Univariate analysis revealed a significant relationship between EAT and VAT that was higher in postmenopausal women with MS, 544.2 ± 122.9, versus those without MS, 363.6 ± 162.3 mm^2^; *p* = 0.03

ACS: acute coronary syndrome, BMI: body mass index, CAD: coronary artery disease, CT: computed tomography, EAT: epicardial adipose tissue, EFV: epicardial fat volume, MCT: multislice computed tomography, MDTM: multidetector computed tomography, MRI: magnetic resonance imaging, MS: metabolic syndrome, PCI: percutaneous intervention, TTE: transthoracic echocardiogram, and VAT: visceral adipose tissue.

**Table 3:** Studies of therapeutic measures to decrease the epicardial fat volume.

Author (reference)	Methodology	Conclusions
Park et al. [157]	Retrospective study in 145 patients who underwent PCI and coronary angiography scheduled for 6 to 8 months later; they underwent two-dimensional TTE in two stages; 82 patients received atorvastatin (20 mg) and 63 patients received simvastatin/ezetimibe (10/10 mg)	The use of statins, particularly atorvastatin, is associated with a reduction in the volume of EAT in patients with CAD; EAT change was 0.47 ± 0.65 mm in the atorvastatin group versus 0.12 ± 0.52 mm in the simvastatin/ezetimibe group; *p* = 0.001; multivariate analysis: atorvastatin group: OR: 0.509; 95% CI: 0.162–0.855; *p* = 0.005

Sacks et al. [158]	Study in 55 patients (12 controls) with CAD, MS, or DM who underwent open heart surgery for fat sample acquisition; genetic analysis was performed by RT-PCR; 7 diabetic patients received pioglitazone 25 mg for 24 months (average)	The use of pioglitazone in patients with coronary artery disease and type 2 DM was associated with a decrease in the genetic expression of proinflammatory and anti-inflammatory cytokines in EAT

Lima-Martínez et al. [159]	Intervention pilot study for 24 weeks in 26 type 2 diabetic patients with HbA1c ≥ 7% on metformin monotherapy; those who met the inclusion criteria received metformin 1000 mg/10 mg sitagliptin and underwent two-dimensional TTE	The addition of sitagliptin to metformin therapy produces a rapid decline in the volume of EAT, thus serving as a noninvasive method (measured by ultrasound) of change in visceral fat during pharmacological interventions (before: 9.98 ± 2.63; after: 8.10 ± 2.11 mm; *p* = 0.001)

Elisha et al. [160]	Randomized pilot study intervention for 6 months in 56 patients (36 treated with insulin detemir and 20 with insulin glargine) who underwent two-dimensional TTE	The use of insulin detemir yielded a reduction in the volume of EAT and less fat gain in comparison with the use of insulin glargine (detemir, −1.7 ± 0.52 mm, versus glargine, −1.1 ± 1.6 mm; *p* < 0.05)

Kim et al. [161]	Study in 24 obese patients who underwent a 12-week supervised exercise training program (60–70% of the maximal heart rate, 60 min/day, 3 days/wk) besides two-dimensionally guided M-mode TTE	The aerobic training significantly reduced the thickness of the EAT, which was also associated with a decrease in visceral adipose tissue (8.11 ± 1.64 versus 7.39 ± 1.54 mm before and after exercise training, resp.; *p* < 0.001)

CAD: coronary artery disease, DM: diabetes mellitus, EAT: epicardial adipose tissue, Hb: hemoglobin, MS: metabolic syndrome, PCI: percutaneous intervention, RT-PCR: reverse transcription polymerase chain reaction, and TTE: transthoracic echocardiogram.
